# Strength in quality: Myosteatosis as a predictor of survival in advanced hepatocellular carcinoma

**DOI:** 10.1002/ueg2.12645

**Published:** 2024-08-02

**Authors:** Claudia Campani, Annalisa Berzigotti

**Affiliations:** ^1^ Internal Medicine and Liver Unit University Hospital Careggi University of Florence Florence Italy; ^2^ Department of Experimental and Clinical Medicine University of Florence Florence Italy; ^3^ Department of Visceral Surgery and Medicine Inselspital Berne University Hospital University of Berne Bern Switzerland

Hepatocellular carcinoma (HCC) prognosis remains challenging due to late‐stage diagnoses, compounded by the absence of biomarkers to predict treatment response. Therapeutic decisions are guided by the Barcelona Clinic Liver Cancer (BCLC) classification, which stratifies patients based on tumor burden, liver function, and performance status, aiming to optimize treatment outcomes by considering the performance of the individual patient.^1^ There is a need for objective and measurable data that is able to assess it. Analysis of body composition on CT or MRI may offer us with potential prognostic imaging biomarkers. Sarcopenia, defined as a reduced skeletal muscle mass and function, has been recognized as such a biomarker. Sarcopenia contributes to a worse prognosis of HCC. While reduced muscle mass is relevant, we should also consider qualitative changes in muscle composition. Myosteatosis is characterised by excessive ectopic fat accumulation either inter‐ or intramuscularly. This is associated with disrupted contractility and reduced muscle function due to lipotoxicity, limited neuromuscular activation, impaired muscle blood flow, and increased local inflammation.^2^ Studies evaluating the impact of myosteatosis in patients with advanced HCC are limited. A study involving 1257 HCC patients showed that myosteatosis was associated with mortality, independent of cancer stage or Child‐Pugh class.^3^ HCC patients with myosteatosis undergoing liver resection had a reduced overall survival (OS) and higher rates of postoperative complications.^4^ Moreover, baseline presence and treatment onset of myosteatosis contribute to worse OS, progression‐free survival, and poorer disease control rate in patients with HCC treated with immune‐checkpoint inhibitors.^5^ Data on the role of myosteatosis in patients treated with intra‐arterial therapy are controversial.^6^, ^7^

In this issue of the *United European Gastroenterology Journal*, Surov et al. described muscle quality as a prognostic factor for HCC patients treated with selective internal radiation therapy (SIRT) + sorafenib; namely, myosteatosis identified a group of patients with poor prognosis after SIRT + sorafenib, but not after sorafenib alone.^8^ In a previous study, the same authors demonstrated that skeletal muscle index (as indicator of sarcopenia), nor visceral, subcutaneous and total adipose tissue predicted OS in patients palliatively managed with SIRT + sorafenib.^9^

The article in the UEG Journal focuses on myosteatosis and is consistent with the finding that skeletal muscle quality, rather than muscle mass or adipose tissue, predicts prognosis. Presence of myosteatosis was associated with a shorter OS in patients treated with SIRT + sorafenib in contrast to sorafenib alone. The authors do not provide a specific explanation for this difference, but it could be hypothesized that myosteatosis, being the consequence of insulin‐resistance and chronic systemic inflammation, identifies patients with lower functional reserve. Alternatively, the effect of myosteatosis on the prognosis in the sorafenib group could be underestimated due to an insufficient sample size. Indeed, a recent study evaluating the role of myosteatosis in 245 HCC patients treated with sorafenib demonstrated that myosteatosis may play a prognostic role in predicting OS.^10^ Differences in population may contribute to the findings as the latter study enrolled Asian patients, whereas the former study predominantly enrolled patients from Western countries.

The authors introduce the albumin‐myosteatosis gauge for the first time in the context of HCC, previously associated with OS in metastatic colorectal and pancreatic cancer patients undergoing first‐line chemotherapy.^11^, ^12^ Low values of albumin‐myosteatosis gauge were independently associated with a worse OS in the SIRT + sorafenib treated patients. However, HCC arises in 80% of cases on a background of cirrhosis with varying degrees of liver function, which can independently influence albumin production regardless of the presence of myosteatosis and pro‐inflammatory cytokines like IL‐1, IL‐6 and TNF‐alpha, which negatively modulate albumin production. Therefore, this parameter needs evaluation in larger cohorts of patients with cirrhosis and with varying levels of liver function, where the assessment of sarcopenia and myosteatosis should be available.

The authors also present results on the predictive role of myosteatosis and the albumin gauge based on HCC etiology. Myosteatosis has been described as a predictor of OS in patients with metabolic liver disease or chronic alcohol consumption related HCC, rather than chronic viral infection. As stressed by the authors, these results must be interpreted with caution, due to the limited number of patients included in this sub‐analysis and as currently no evidence suggesting that etiology may predict response and prognosis of patients is available. However, these results may directly reflect the pathophysiology of myosteatosis. Indeed, ethanol can impair muscle protein homeostasis and promote fat accumulation, and in MASLD, myosteatosis has been described in both early and advanced stage of disease, likely in response to insulin‐resistance.^13^

To conclude, imaging markers of muscle quality and quantity, particularly myosteatosis, may predict prognosis of patients with advanced HCC. Patients with myosteatosis may benefit from nutrition optimization and aerobic and resistance training as it may contribute to a better physical fitness and overall prognosis in HCC.
